# Supplementary material for: Pareto-based evaluation of national responses to COVID-19 pandemic shows that saving lives and protecting economy are non-trade-off objectives
Source: Sci Rep. 2021 Jan 28;11:2425. doi: 10.1038/s41598-021-81869-2 (PMC7844048; doi:10.1038/s41598-021-81869-2)
Supplement: Supplementary file 1 — Supplementary Information. [file 41598_2021_81869_MOESM1_ESM.pdf]

Supplementary Information

featuring the article

"Pareto-based evaluation of national responses to COVID-19 pandemic shows that saving lives and protecting economy are non-trade-off objectives"

by M. Kochańczyk & T. Lipniacki

Scientific Reports (2020/2021)

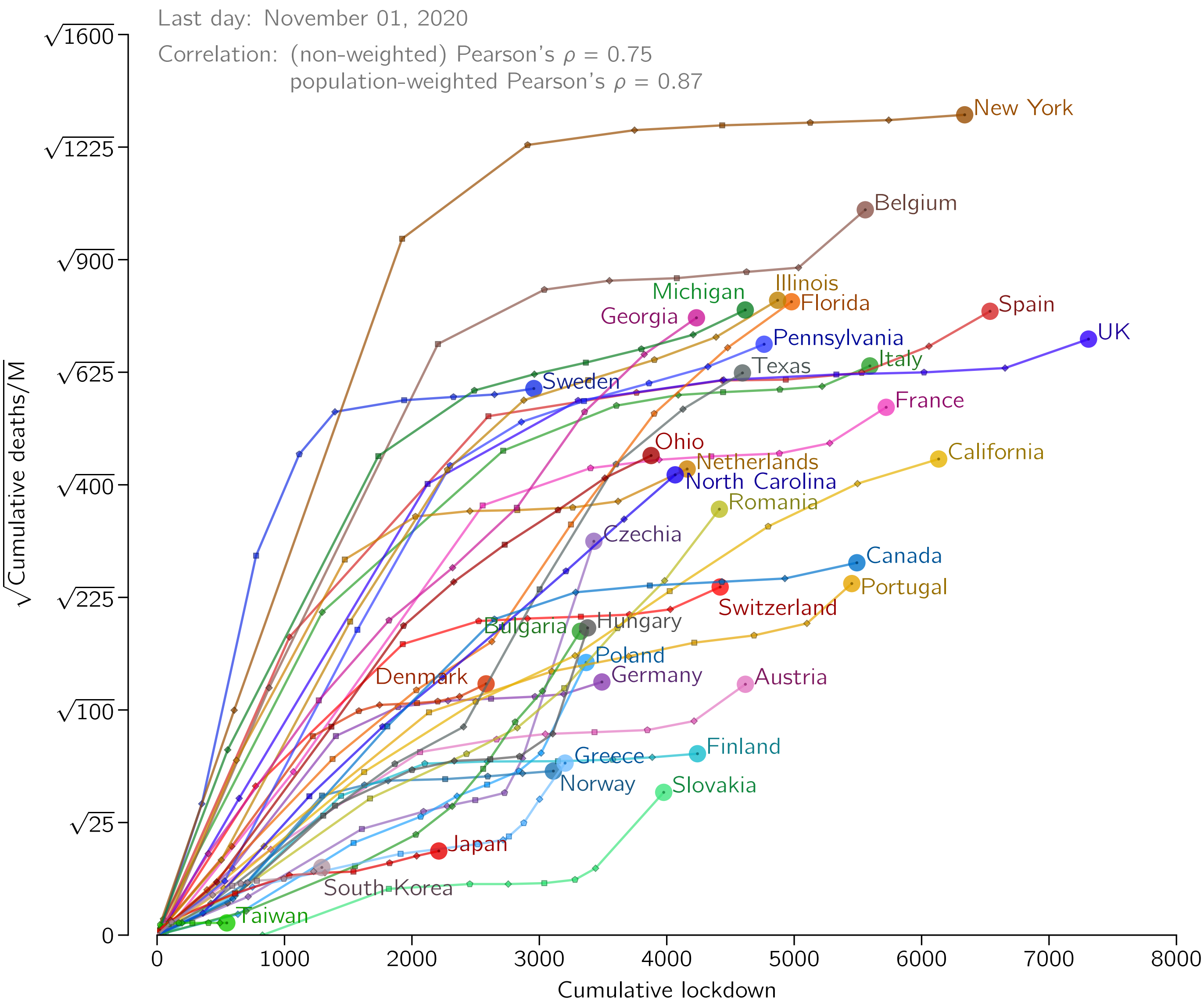

Supplementary Figure S1: Time trajectories of countries and US states analyzed in Fig. 1 in the main text in the cumulative lockdown *vs.* square root of the population-normalized cumulative number of COVID-19-assigned deaths plane. Trajectories begin in February and end on November 1, 2020, and have a one-month resolution.
